# Association of Organochlorine Pesticides With Genetic Markers of Endoplasmic Reticulum Stress in Type 2 Diabetes Mellitus: A Case–Control Study Among the North-Indian Population

**DOI:** 10.3389/fendo.2022.841463

**Published:** 2022-03-16

**Authors:** Neha Tawar, Basu Dev Banerjee, Sri Venkata Madhu, Vivek Agrawal, Sanjay Gupta

**Affiliations:** ^1^ Department of Biochemistry, University College of Medical Sciences (UCMS) and Guru Teg Bahadur (GTB) Hospital (University of Delhi), Delhi, India; ^2^ Department of Endocrinology and Metabolism, UCMS and Guru Teg Bahadur (GTB) Hospital (University of Delhi), Delhi, India; ^3^ Department of Surgery, UCMS and Guru Teg Bahadur (GTB) Hospital (University of Delhi), Delhi, India

**Keywords:** OCP, T2DM, normal glucose tolerance, inflammation, ER stress, visceral adipose tissue

## Abstract

**Background:**

Organochlorine pesticides (OCPs) have been long linked to type 2 diabetes mellitus (T2DM); however, this relation at the molecular level has not been explored yet. Endoplasmic reticulum (ER) stress and pro-inflammatory pathways are considered vital ones in the pathogenesis of T2DM. We aimed to investigate the existence of any association between OCPs, ER stress, and pro-inflammatory pathways in subjects with known T2DM.

**Methods:**

Seventy subjects each with T2DM and normal glucose tolerance were recruited from the surgery department. Their visceral adipose tissue was collected intraoperatively. OCP concentration, ER stress, and pro-inflammatory markers were analyzed and compared between two study groups.

**Results:**

We found 18 OCPs and their metabolites in visceral adipose tissue samples of study participants. The levels of δ-HCH, heptachlor, endrin, and p,p′DDT were significantly higher in the T2DM group and were also positively correlated with fasting and postprandial plasma glucose levels (p < 0.01). We observed a positive association of δ-HCH (p < 0.01), heptachlor (p < 0.05), and endrin (p < 0.05) with central adiposity and ER stress markers. However, we failed to establish the correlation of OCPs with any of the pro-inflammatory markers.

**Conclusion:**

The existence and simultaneous complex correlation of OCPs with ER stress may explain their role in the pathogenesis of T2DM, revealing the persistence of the gene–environment interaction in the etiology of T2DM.

## Introduction

Exposure to environmental toxicants such as organochlorine pesticides (OCPs) has been strongly associated with type 2 diabetes mellitus (T2DM) (1–6). Their property of persistence and bioaccumulation in the environment has generated a surge of global interest (7). Due to their lipophilic nature, they remain stored in the adipose tissue without disintegration for a long time (8); in consequence, even a minimal continuous exposure over time can still lead to potential health hazards (9). OCPs are the most investigated insecticides linked with obesity in humans (10). India is among one of the top producers and consumers of OCPs (11, 12), and their exposure might be a possible risk factor for higher vulnerability for developing T2DM (3).

Adipose tissue, which is considered an energy storage site, has also been associated with several adverse metabolic consequences, providing visceral adipose tissue more physiological relevance, as it is a leading determinant of insulin resistance and excessive inflammatory state (13). With the development of obesity, the adipose tissue undergoes some molecular and cellular adaptations with a significant generation of pro-inflammatory molecules such as tumor necrosis factor α (TNFα) and interleukin 6 (IL-6) (14). Chronic inflammation may be a consequence of endoplasmic reticulum (ER) stress and/or trigger the same (15), which ensures a self-perpetuating cycle. Chronic ER stress, which is considered to be a vital one in diabetes pathogenesis (16, 17), leads to a self-protective mechanism to maintain cellular viability and function by reducing misfolded and unfolded protein load by unfolded protein response (UPR) (18). Upregulated ER stress has been observed in the adipose tissue of various obese and lean insulin-resistant animals and humans (19–21).

In spite of major advancements in the acknowledgement of the pathophysiology of T2DM, the mechanisms are still incompletely understood and its susceptibility might be explained by the complex gene–environment interaction (22). Although the previous literature supports the evidence for the complex interplay of ER stress and inflammation in chronic metabolic disorders, there is a dearth of studies showing the interconnectivity of these environmental contaminants with such cellular stress mechanisms. Hence, the present study was conducted in order to evaluate the possible synergy of OCP compounds with pro-survival and pro-apoptotic ER stress and inflammatory pathways at the molecular level in human subjects with T2DM.

## Methodology

This was a case–control study carried out in the Department of Surgery and Biochemistry, University College of Medical Sciences (UCMS), and Guru Teg Bahadur (GTB) Hospital, Delhi. Ethical clearance (certificate no. IHEC-UCMS 08112015) was obtained from the Institutional Ethics Committee for Human Research, UCMS, and GTB Hospital, Delhi, before initiating the study. The patient information sheet was explained to each subject, and their written consent was obtained. The study was conducted between the periods of January 2016 to January 2020.

### Study Subjects

The study included 70 subjects each with known T2DM and normal glucose tolerance (NGT) subjects attending the OPD of the surgery department for their elective abdominal surgeries for clinical conditions such as cholecystectomy, appendectomy, and hernia. Subjects with life-threatening cardiovascular disease, known pancreatic disease, and Cushing syndrome as well as females with pregnancy and known polycystic ovarian syndrome were excluded from the study.

The T2DM subjects were categorized based on clinical guidelines set by the American Diabetes Association (ADA) (23), while a standardized 75-g oral glucose tolerance test was performed for identifying subjects with NGT (23). Both the groups were matched based on their gender, age, and BMI.

### Anthropometric Measurements

A detailed questionnaire was used to record the demographical and clinical characteristics of the recruited subjects. Demographical characteristics including age, gender, body mass index (weight (kg)/height (m^2^)), pulse rate, blood pressure, diet pattern, socioeconomic status (24), and any other medical history were documented for each subject.

### Sample Collection and Storage

The visceral adipose tissue sample weighing about 5 g was collected intraoperatively in a sterile container and cryopreserved in liquid nitrogen followed by their storage at -80°C for estimation of OCP levels and gene expression levels. Also, blood samples were collected for the estimation of fasting and postprandial plasma glucose.

### Organochlorine Pesticide Analysis

Pesticide extraction for each sample was done in triplicates by using the method given by Bush et al. (25). Briefly, 1 g of finely chopped visceral adipose tissue was shaken in a flask with 1:2 parts of acetone (Merck, Kenilworth, NJ, USA) and n-hexane (Merck, USA) at 100–120 rpm at room temperature for 4 to 5 h on a mechanical shaker. The solvent was collected and the extraction procedure was repeated twice using the same process, and the fractions were added to the previous solvent fractions. The cleanup of the collected solvent fraction was done by adsorption column chromatography using heat-activated Florisil (SRL, Mumbai, India) and anhydrous sodium sulfate (SRL, India). The collected n-hexane was evaporated by a vacuum rotary evaporator and reconstituted in n-hexane to 1-ml quantity. Quantification of organochlorine residue levels was done using a gas chromatograph (PerkinElmer Clarus 500) equipped with a Ni63 electron capture detector. The sample (1 µl) was automated injected at 170°C with a hold of 1 min. The temperature was raised at the rate of 5°C/min to 225°C and the time for the hold was 5 min, and then to 275°C at the rate of 6°C/min with a 15-min hold. Nitrogen gas with a flow rate of 35 ml/min was used as a carrier. The limit of detection for each pesticide was 4 pg/ml. The adipose tissue samples were analyzed for the following organochlorine residues: α-hexachlorocyclohexane (α-HCH), β-HCH, γ-HCH, δ-HCH, heptachlor, aldrin, heptachlor epoxide B (HTEB), heptachlor epoxide A (HTEA), endosulfan I, endosulfan II, dieldrin, endrin, methoxychlor, o,p′-dichlorodiphenyldichloroethylene (o,p′-DDE), p,p′-DDE, p,p′-dichlorodiphenyldichloroethane (p,p′-DDD), o,p′-dichlorodiphenyltrichloroethane (o,p′-DDT), and p,p′-DDT. A quantitative analysis of pesticide residues in each sample was done by comparing the peak area with those obtained from a chromatogram of a mixed OCP standard (AccuStandard, New Haven, CT, USA) of known concentrations. The concentration of OCPs in samples has been presented in ng/gm (ppb) wet weight.

### Gene Expression Analysis at the Transcriptional Level

The mRNA expression of protein kinase R-like ER kinase (PERK), activating transcription factor (ATF-4), C/EBP homologous protein (CHOP), inositol-requiring enzyme-1α (IRE-1α), X box-binding protein (spliced) (XBP-1s), glucose-regulated protein-78 (GRP-78), TNFα, and IL-6 were analyzed by quantitative real-time PCR (qPCR) in triplicates along with negative control.

Total RNA extraction from visceral adipose tissue was done by using the TRIzol reagent (Invitrogen, Thermo Fisher Scientific, Waltham, MA, USA) standard method as per the manufacturer’s protocol. The quality and concentration of total RNA were measured spectrophotometrically on a NanoDrop spectrophotometer (ND2000, Thermo Fisher Scientific, USA). Total RNA (1 µg) was used for complementary DNA synthesis using an iScript cDNA synthesis kit (Bio-Rad, Hercules, CA, USA) as per the manufacturer’s protocol. The NCBI tool, Primer BLAST, was used to design the primers of genes of interest. The Sso EvaGreen Supermix (Bio-Rad, USA) (dNTPs, Sso7d fusion polymerase, MgCl_2_, EvaGreen dye, stabilizers) was used for the reaction mixture of qPCR. The concentration of 10 pmol of primer was used for each qPCR reaction (primer sequences presented in **Table 1**). The PCR amplification was performed on a CFX Connect Real-Time PCR Detection System (Bio-Rad, USA). The amplification cycle included initial denaturation at 95°C for 1 min followed by 40 cycles of denaturation at 95°C for 30 s and annealing at 60°C for 45 s and lastly extension at 72°C for 30 s. β-Actin was used as the gene normalizer. The gene expression was determined by the 2-ΔΔCt method (26).

**Table 1:** Primer sequences of specific genes.

Genes	Primer sequence
**PERK**	Forward 5′ TGTCGCCAATGGGATAGTGACGA 3′
	Reverse 5′ AATCCGGCTCTCGTTTCCATGTCT 3′
**ATF-4**	Forward 5′ GGGAGTTGGCTTCTGATTCTCA 3′
	Reverse 5′ ATCAAGTCCCCCACCAACAC 3′
**CHOP**	Forward 5′ AAACAGGCATCAGACCAGCTT 3′
	Reverse 5′ CTGCCATCTCTGCAGTTGGA 3′
**IRE-1α**	Forward 5′ ACACCATCACCATGTACGACACCA 3′
	Reverse 5′ ATTCACTGTCCACAGTCACCACCA 3′
**XBP-1s**	Forward 5′ TGCTGAGTCCGCAGCAGGTG 3′
	Reverse 5′ GCTGGCAGGCTCTGGGGAAG 3′
**GRP-78**	Forward 5′ CATCACGCCGTCCTATGTCG 3′
	Reverse 5′ CGTCAAAGACCGTGTTCTCG 3′
**IL-6**	Forward 5′ AAAGATGTAGCCGCCCCAC 3′
	Reverse 5′ AGGCAAGTCTCCTCATTGAATCC 3′
**TNF α**	Forward 5′ CCCAGGCAGTCAGATCAT 3′
	Reverse 5′ TCAGCTCCACGCCATT 3′
**β-Actin**	Forward 5′ GTCTTCCCCTCCATCGT 3′
	Reverse 5′ CGTCGCCCACATAGGAAT 3′

### Gene Expression Analysis at the Translational Level

The total proteins from 100 mg visceral adipose tissue samples were isolated by radioimmunoprecipitation buffer (G-Biosciences, St. Louis, MO, USA) followed by their quantification using a bicinchoninic acid protein assay kit (G-Biosciences, USA). In brief, 30 µg denatured protein was resolved on 10% Tris–glycine gel and transferred to a polyvinylidene fluoride membrane (Bio-Rad, USA). The non-specific binding sites on the membrane were blocked with 5% skimmed milk. The membrane was incubated with corresponding primary antibodies (Abbkine, USA) at 1:700 dilutions overnight at 4°C, and after washing the membrane was treated with an HRP-conjugated secondary antibody (Abbkine, USA) for 2 h. The detection of signals was recorded by the use of an ECL Reagent Kit (Thermo Fisher Scientific, USA) in the gel imaging system (Thermo Fisher Scientific ECL Imager). The protein band density was measured and compared using the ImageJ software.

### Statistical Analysis

The data for continuous variables were expressed as mean ± standard deviation. Different variables between cases and controls were compared by the independent t-test. The Mann–Whitney U test was used to compare the pesticide level between cases and controls. The adjusted odds ratio (OR) with 95% confidence interval was calculated for defining the risk of T2DM by OCPs. The Spearman correlation matrix was used to analyze data for biochemical variables with pesticides, and gene–environment interaction. p values <0.05 were considered statistically significant. Collected data were analyzed using IBM SPSS software (version 21).

## Results

### Demographic Characteristics of Study Groups

The demographic characteristics of the study subjects are presented in **Table 2**. The study subjects in both T2DM and NGT groups shared similar anthropometric characteristics with a comparable proportion of males and females (p = 0.856). The two groups did not share a significant age difference (p = 0.409). There were no significant differences in BMI (p = 0.291) between the study groups; however, their waist circumference differs significantly (p ≤ 0.001). There was no significant difference observed in blood pressure between the groups (p = 0.102). The case group shared a higher history of familial diabetes (p = 0.003) as compared to controls. The socioeconomic status also differed significantly between the two groups (0.018); the subjects with T2DM belonged to a higher socioeconomic group compared to subjects of the control group.

**Table 2:** Demographic characteristics of the T2DM and NGT groups.

Parameters	NGT (N = 70)	T2DM (N = 70)	p-value
**Gender (male/female)**	23/47	21/49	0.856
**Age (years)**	43.65 ± 9.22	44.9 ± 8.61	0.409
**BMI (kg/m^2^)**	24.3 ± 3.3	24.9 ± 3.3	0.291
**Waist circumference (cm)**	88 ± 7.2	95.3 ± 10	0.000^***^
**BP diastolic/systolic (mmHg)**	80.4 ± 6.2/123.5 ± 8.1	81.5 ± 6.1/126.1 ± 10.9	0.102
**Positive family history of T2DM**	16	34	0.003^**^
**Socioeconomic status (I/II/III/IV/V)**	0/0/4/16/50	0/8/7/14/41	0.018^*^

Values presented as mean ± SD; *p ≤ 0.05; **p ≤ 0.01; ***p ≤ 0.001.

### Organochlorine Pesticide Analysis

**Figure 1:**
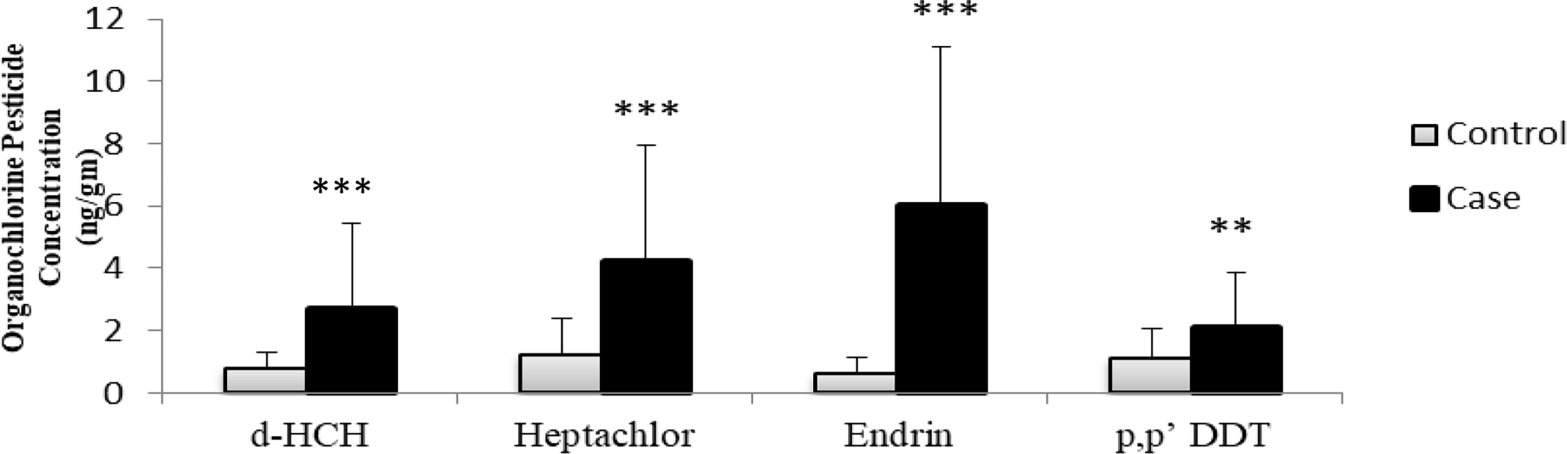
Comparison of OCP levels (ng/gm) in the T2DM (N = 70) and NGT (N = 70) groups. **p ≤ 0.01; ***p ≤ 0.001.

The analysis of visceral adipose tissue samples disclosed the presence of various OCPs and their metabolites. By comparing the OCP levels, we found that every pesticide concentration was higher in the T2DM group as compared to the NGT group (**Table 3**); however, δ-HCH (p ≤ 0.001), heptachlor (p ≤ 0.001), endrin (p ≤ 0.001), and p,p′-DDT (p = 0.002) levels were significantly higher in T2DM patients (**Figure 1**). Multivariable binary logistic regression, adjusted for confounding factors such as age, gender, BMI, and family history of T2DM, revealed that of these four pesticides, δ-HCH (p = 0.003) and endrin (p ≤ 0.001) were positively associated with the risk of T2DM (**Table 4**).

**Table 3:** Comparison of visceral adipose tissue OCP levels between the T2DM and NGT groups.

Organochlorine pesticides (ng/gm)	Controls (mean ± SD) N = 70	Cases (mean ± SD) N = 70	p-value
**α-HCH**	0.41 ± 1.07	0.90 ± 2.45	0.131
**β-HCH**	0.89 ± 2.54	1.25 ± 5.56	0.624
**γ-HCH**	0.86 ± 1.88	1.17 ± 2.88	0.451
**δ-HCH**	0.79 ± 0.50	2.70 ± 2.73	0.000^***^
**Heptachlor**	1.22 ± 1.17	4.20 ± 3.74	0.000^***^
**Aldrin**	0.57 ± 1.77	0.65 ± 2.34	0.830
**HTEB**	0.12 ± 0.45	0.36 ± 0.93	0.053
**HTEA**	1.81 ± 2.99	1.81 ± 4.70	0.244
**o,p′-DDE**	1.07 ± 2.71	1.82 ± 6.48	0.378
**Endosulfan I**	1.15 ± 4.23	1.77 ± 4.25	0.391
**Dieldrin**	0.09 ± 0.64	0.38 ± 1.09	0.062
**p,p′-DDE**	0.53 ± 2.19	1.11 ± 4.80	0.368
**Endrin**	0.60 ± 0.56	6.01 ± 5.08	0.000^***^
**Endosulfan II**	0.37 ± 1.89	0.61 ± 2.20	0.062
**p,p′-DDD**	1.74 ± 3.21	2.53 ± 3.80	0.193
**o,p′-DDT**	0.35 ± 0.99	0.72 ± 1.48	0.121
**Methoxychlor**	0.23 ± 1.22	0.62 ± 1.46	0.105
**p,p′-DDT**	1.13 ± 0.94	2.09 ± 1.77	0.000^***^

SD, standard deviation.

***p ≤ 0.001.

**Table 4:** The risk of development of T2DM with visceral adipose tissue accumulation of OCPs.

Organochlorine pesticides	Adjusted odds ratio	95% CI	p-value
**δ-HCH**	3.835	1.581–9.302	0.003^**^
**Endrin**	4.127	1.905–8.838	0.000^***^
**Heptachlor**	1.309	0.928–1.846	0.125
**p,p′-DDT**	1.149	0.610–2.165	0.666

Adjusted for age, gender, BMI, and family history of T2DM.

CI, confidence interval.

**p ≤ 0.01; ***p ≤ 0.001.

### The Correlation of Glycemic and Anthropometric Markers With OCP Levels

**Figure 2:**
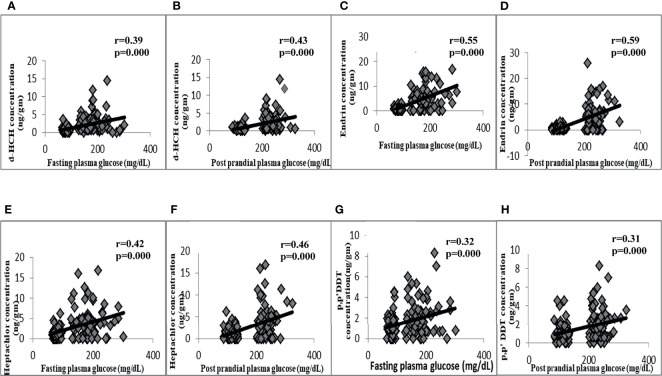
Correlation of OCPs with plasma glucose indices. Positive correlation of fasting plasma glucose with d-HCH **(A)**, endrin **(C)**, heptachlor **(E)**, and p,p’DDT **(G)**. Positive correlation of postprandial plasma glucose with d-HCH **(B)**, endrin **(D)**, heptachlor **(F)**, and p,p’DDT **(H)**.

The correlation analysis also revealed the significant positive correlation of fasting and postprandial plasma glucose with δ-HCH, heptachlor, endrin, and p,p′-DDT (**Figure 2**). The waist circumference was also found to be positively correlated with δ-HCH (r = 0.242, p = 0.004), heptachlor (r = 0.207, p = 0.014), and endrin (r = 0.211, p = 0.012).

### ER Stress and Pro-Inflammatory Marker Gene Expression

**Figure 3:**
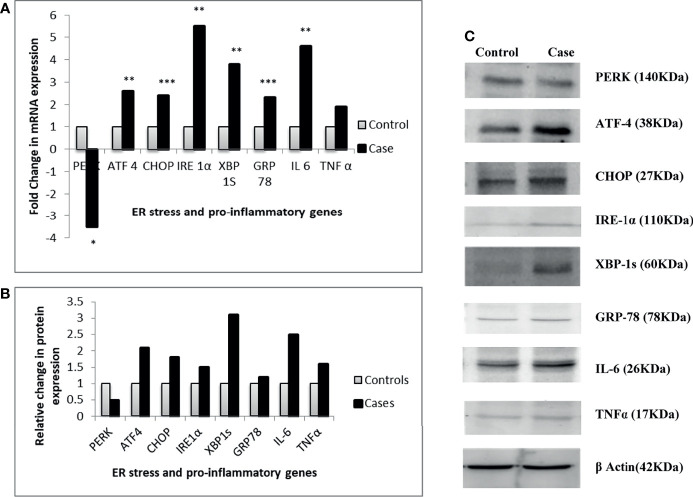
Relative gene expression of ER stress and pro-inflammatory genes between thetwo groups. **(A)** Relative change in transcriptional expression of the genes. **(B)** Relative change in translational expression of the genes. **(C)** Western blot images of the genes. *p ≤ 0.05; **p ≤ 0.01; ***p ≤ 0.001.

The parallel transcriptional and translational gene expression analysis revealed that all the ER stress markers except PERK were upregulated in visceral adipose tissue of T2DM patients as compared to NGT. A higher expression of both the pro-inflammatory markers was also observed in the T2DM group. The mRNA expressions of ATF-4, CHOP, IRE-1α, XBP-1s, and GRP-78 were 2.6-, 2.4-, 5.5-, 3.8-, 2.3-fold upregulated respectively in the T2DM group compared with the NGT group, whereas PERK was found to be 3.5-fold downregulated. Similarly, the mRNA expression of IL-6 and TNFα was found to be 4.6- and 1.9-fold significantly higher in T2DM subjects compared to NGT subjects, respectively. The protein expression of the respective genes was found to be in synchronization with their transcriptional expression (**Figure 3**).

### The Gene–Gene Interaction

Interestingly, the mRNA expression of all the ER stress markers significantly and positively correlated among themselves and with both the pro-inflammatory markers (**Table 5**).

**Table 5:** Correlation matrix showing the gene–gene interaction among ER stress and pro-inflammatory markers in visceral adipose tissue.

Gene	PERK	ATF-4	CHOP	IRE-1α	XBP-1s	GRP-78	IL-6	TNFα
	r	r	r	r	r	r	r	r
**PERK**	1	0.000	0.274^*^	0.091	0.249^*^	0.272^*^	0.409^***^	0.480^***^
**ATF-4**	0.000	1	0.404^**^	0.188	0.279^*^	0.458^***^	0.176	0.245^*^
**CHOP**	0.274^*^	0.404^**^	1	0.379^**^	0.471^***^	0.571^***^	0.600^***^	0.417^***^
**IRE-1α**	0.091	0.188	0.379^**^	1	0.637^***^	0.212	0.443^***^	0.250^*^
**XBP-1s**	0.249^*^	0.279^*^	0.471^***^	0.637^***^	1	0.279^*^	0.420^***^	0.398^**^
**GRP-78**	0.272^*^	0.458^***^	0.571^***^	0.212	0.279^*^	1	0.283^*^	0.390^**^
**IL-6**	0.409^***^	0.176	0.600^***^	0.443^***^	0.420^***^	0.283^*^	1	0.580^***^
**TNF α**	0.480^***^	0.245^*^	0.417^***^	0.250^*^	0.398^**^	0.390^**^	0.580^***^	1

r, correlation coefficient.

*p ≤ 0.05; **p ≤ 0.01; ***p ≤ 0.001.

### The Correlation of Glycemic and Anthropometric Markers With ER Stress and Pro-Inflammatory Gene Expression

By correlating the studied genes with fasting and postprandial plasma glucose, all genes except PERK and TNFα were found to be significantly positively correlated with plasma glucose indices. Interestingly, the weight of the study subjects was found to be positively correlated with mRNA expressions of CHOP and GRP-78. Waist circumference showed a significant positive correlation with ATF-4, CHOP and GRP-78, and BP (systolic and diastolic) with IRE-1α, and XBP-1s (**Table 6**).

**Table 6:** Correlation matrix showing the association of mRNA expression of ER stress and pro-inflammatory genes with anthropometric and clinical variables.

Variables	Δ Ct PERK	Δ Ct ATF-4	Δ Ct CHOP	Δ Ct IRE-1α	Δ Ct XBP-1s	Δ Ct GRP-78	Δ Ct IL-6	Δ Ct TNFα
	r	r	r	r	r	r	r	r
**Height**	0.11	0.14	0.4	0.24	0.15	0.4	0.32	0.2
**Weight**	0.08	0.32	0.5^*^	0.31	0.15	0.53^*^	0.27	0.32
**BMI**	0.02	0.31	0.31	0.19	0.05	0.38	0.07	0.28
**Waist circumference**	0.02	0.49^*^	0.64^**^	0.38	0.26	0.66^***^	0.37	0.43
**BP systolic**	0.06	0.59^**^	0.42	0.64^**^	0.66^**^	0.36	0.42	0.3
**BP diastolic**	0.02	0.44	0.23	0.61^*^	0.58^**^	0.14	0.28	0.21
**Fasting plasma glucose**	-0.29	0.40^***^	0.41^***^	0.43^***^	0.46^***^	0.32**	0.35^***^	0.15
**Postprandial plasma glucose**	-0.23	0.44^***^	0.42^***^	0.40^***^	0.50^***^	0.37^***^	0.29^*^	0.17

r, correlation coefficient.

*p ≤ 0.05; **p ≤ 0.01; ***p ≤ 0.001.

### The Gene–Environment Interaction

A significant positive correlation between transcriptional ER stress expression and OCPs was observed in this study. δ-HCH was positively correlated with the expression of ATF-4 (p = 0.010), IRE-1α (p = 0.030), and XBP-1s (p = 0.038) genes. Heptachlor was found to be positively correlated with IRE-1α (p = 0.044) and XBP-1s (p = 0.006) genes, whereas endrin was positively correlated with ATF-4 (p = 0.025), CHOP (p = 0.017), IRE-1α (p = 0.039), XBP-1s (p = 0.003), and GRP-78 (p = 0.020) genes (**Table 7**). However, a significant correlation of genes with p,p′-DDT was not observed in our study.

**Table 7:** Correlation matrix showing gene–environment interaction between ER stress markers and OCPs.

Genes/OCPs	δ-HCH	Heptachlor	Endrin
	r	r	r
**ATF-4**	0.284^**^	0.039	0.247^*^
**CHOP**	0.175	0.219	0.286^*^
**IRE-1α**	0.261^*^	0.243^*^	0.250^*^
**XBP-1s**	0.250^*^	0.328^**^	0.353^**^
**GRP-78**	0.167	0.145	0.280^*^

r, correlation coefficient.

*p ≤ 0.05; **p ≤ 0.01.

## Discussion

Our study demonstrated the presence of several OCPs and their metabolites in the visceral adipose tissue, of which δ-HCH, heptachlor, endrin, and p,p′-DDT were found to be significantly higher in the T2DM group as compared to the NGT group. These pesticides were positively associated with central adiposity, linking their obesogenic role in the etiology of T2DM. Nevertheless, the odds ratios of δ-HCH and endrin were significantly associated with the occurrence of T2DM. Our study is the first to show the interplay of ER stress with OCP compounds in T2DM and establish the possible cross talk between genetics and the environment. We found endrin to be correlated with all ER stress genetic markers except the PERK gene; however, we failed to establish the synergistic relation between pro-inflammatory markers and OCPs.

Upregulation of ER stress and pro-inflammatory markers in the visceral adipose tissue and their significant positive correlation with fasting and postprandial plasma glucose have been found in our study, which indicates that both of these pathways might be considered as an attractive target for T2DM.

Discussing the part of OCPs in diabetes, numerous epidemiological studies have associated OCP exposure with increased risks of obesity and/or T2DM (27–30). Due to their lipophilic nature, many pesticides tend to get accumulated in adipose tissue depots and therefore may interrupt the function of adipose tissue and promote obesity and T2DM (30, 31). The role of OCPs as endocrine-disrupting chemicals is also well known (32). In this study, we detected various OCP compounds in the visceral adipose tissue, but only four pesticides, δ-HCH, heptachlor, endrin, and p,p′-DDT, had a mean concentration significantly higher in T2DM subjects as compared to the NGT group. Of these four OCPs, δ-HCH and endrin were found to have a significantly higher relative risk associated with T2DM. These results have never been reported previously in an epidemiological study. However, as per the previous literature, β-HCH was strongly associated with T2DM (2), but δ-HCH has not been linked to T2DM yet, to the best of our knowledge. Heptachlor has been reported to increase the gluconeogenic enzymatic activity in the liver, which in turn upregulates the glucose synthesis from glycogen (33). Kamel et al. uncovered the cumulative effect of heptachlor in the pathogenesis of diabetes (34, 35). Higher serum endrin levels were observed in children with type 1 diabetes (36). Endrin was also found to stimulate lipid accumulation in adipocytes in an *in vitro* study (37). DDT and its metabolites being ubiquitous in the environment have long been linked to endocrine disruption; however, the actual mechanism remains unclear (38). A significant association of visceral adipose tissue levels of DDT with T2DM has also been reported (39). *In vitro* and *in vivo* studies have shown the inhibition of insulin-dependent glucose uptake when treated with an OCP mixture (40).

By the correlation matrix findings, we brought to light the positive correlation of δ-HCH, heptachlor, endrin, and p,p’-DDT with fasting and postprandial plasma glucose. The levels of serum p,p′-DDE were reported to be positively correlated with fasting and postprandial plasma glucose and glycated hemoglobin as well (41). Al-Othman also reported the strong correlation of HCH with homeostatic model assessment for insulin resistance (HOMA-IR) (42); likewise, β-HCH serum levels were found to be elevated in patients with high serum glucose levels (5). Discussing the molecular axis in T2DM, accruing evidence revealed the increased unfolded protein response (UPR)-initiating molecules, PERK and IRE1α, in the adipose tissue of obese humans (19, 20, 43), and human islet cells from T2DM subjects as well (44). Nakatani et al. also showed that ER stress plays a crucial role in the insulin resistance found in diabetes and thus could be a potential therapeutic target for diabetes (45). Our results were consistent with the literature as the markers of the IRE-1α-XBP1-GRP-78 pathway, an early trigger of UPR, were overexpressed in the visceral adipose tissue of T2DM subjects. IRE-1α, a proximal ER stress sensor and central mediator of UPR, upregulates the expression of genes for GRP-78, an ER chaperon *via* splicing of a transcription activator, XBP-1, that ultimately facilitate cellular recovery. GRP-78 also acts as a central regulator for ER stress owing to its role in the induction of stress *via* three transmembrane ER stress sensors through a binding-release mechanism (46).

Considering the PERK-ATF-4-GRP-78 pathway, PERK was found to be downregulated in T2DM subjects in our study, which might be explained by the fact that, during acute or chronic gluco-lipotoxic stresses in secretory cells, pancreatic β cells, and adipocytes, in particular, excessive demand of protein synthesis arises (47). The increased frequency of transcription and translation was observed for the pathway’s downstream markers, ATF-4, mainly responsible for the induction of a pro-apoptotic molecule, CHOP, indicating their activation in the visceral adipose tissue of T2DM subjects. CHOP usually remains expressed at lower levels in unstressed cells; however, under irredeemable ER stress, its expression raises significantly (48) but at the same time its pro-apoptotic effect may be dependent on the corresponding expression of other components of the UPR (49).

ER stress has been positively associated with chronic inflammation in humans as shown in many studies. A study by Lenin et al. in PBMC of T2DM patients as a surrogate cell model demonstrated the elevated ER stress markers and association of these stress molecules with pro-inflammatory markers, TNFα and IL-6 (50). Our results were consistent with previous results as gene expression levels of these two pro-inflammatory markers in the visceral adipose tissue of T2DM subjects were significantly positively correlated with all ER stress markers.

Focusing on the gene expression correlation with blood glucose indices, our study results were consistent with the previously existing data as significant positive correlations between fasting and postprandial plasma glucose and ER stress markers (ATF-4, CHOP, IRE-1α, XBP-1, and GRP-78) and IL-6 were observed. Lenin et al. reported the positive correlations of ATF-4, CHOP, IRE-1α, XBP-1, and GRP-78 with glycated hemoglobin; also later, three markers were closely linked to fasting plasma sugar (50). The serum IL-6 levels have been associated with T2DM by many authors (51, 52), and elevation of this cytokine caused a direct influence on insulin signal intensity (53). However, any considerable association of PERK and TNFα was not found with plasma glucose levels. Being a well-recognized molecular link between obesity, declining insulin action, and eventually the development of T2DM, ER stress enacts in a complex way (54). Early experiments using cell culture and mouse models have demonstrated that excessive adiposity results in chronic ER stress, particularly in the liver and adipose tissue (21). The present study demonstrated the strong correlation of CHOP and GRP-78 with the body weight and waist circumference of the study subjects. Besides, ATF-4 was also significantly correlated with waist circumference. Recent studies have provided evidence that moderate weight loss promotes the amelioration of ER stress in the adipose tissue (20, 55).

Addressing the gene–environment interaction, only a few studies have reported the correlation between several types of insecticides and ER stress. An *in-vitro* study reported the GRP-78 upregulation by endosulfan treatment (56). The existence of a correlation between transcriptional ER stress markers and OCPs in our study suggests some interplay between the two. The results indicate that upregulation of these genes may occur in the presence of a higher concentration of OCP levels in the adipose tissue or its reverse may also be true. Our results demonstrated a positive correlation of δ-HCH with ATF-4, IRE-1α, and XBP-1s, heptachlor with IRE-1α, and XBP-1s, whereas endrin correlated with all ER stress markers except PERK. We did not observe any significant association of OCPs with any of the pro-inflammatory markers. Taken together, these data demonstrate that there might be considerable cross talk between the environmental signaling and cellular pathways. Obesogenic effects of OCPs may contribute to the development of cellular stress which in the long run may be responsible for the pathogenesis of T2DM.

Although there are inadequate data on the underlying mechanisms of OCP toxicity in relation to disease etiology, multiple factors for pollutant exposure and physiological responses also cause some limitations to the study. Concurrently, because of the cross-sectional nature of our work, the study could not reveal any causal relationship between ER stress and T2DM, and for this, we need prospective follow-up studies. In addition, further studies using a larger sample size are needed to identify the association between ER stress and other toxic compounds and confirm their role in the etiology of T2DM.

## Conclusion

The existence of the correlation between OCPs and ER stress markers suggests some interplay between genetics and the environmental factors. The upregulation of genes involved in ER stress may occur in the presence of higher concentration of OCPs levels in the visceral adipose tissue. The study shows the potential role of OCPs in the development of T2DM *via* disrupting the ER stress pathway.

## Data Availability Statement

The raw data supporting the conclusions of this article will be made available by the authors, without undue reservation.

## Ethics Statement

The study involving human participants were reviewed and approved by the Institutional Ethics Committee-Human Research, University College of Medical Sciences. The patients/participants provided their written informed consent to participate in this study.

## Author Contributions

NT: conceptualization, methodology, data curation, writing—original draft preparation. BD: conceptualization, supervision, visualization. SM: supervision, visualization. VA: supervision, visualization, sample collection. SG: supervision, visualization, sample collection. All authors contributed to the article and approved the submitted version.

## Funding

This study was supported by a Junior Research Fellowship provided by the University Grant Commission (no. 132003443).

## Conflict of Interest

The authors declare that the research was conducted in the absence of any commercial or financial relationships that could be construed as a potential conflict of interest.

## Publisher’s Note

All claims expressed in this article are solely those of the authors and do not necessarily represent those of their affiliated organizations, or those of the publisher, the editors and the reviewers. Any product that may be evaluated in this article, or claim that may be made by its manufacturer, is not guaranteed or endorsed by the publisher.
